# Associations of the Planetary Health Diet Index with diet quality, environmental footprints, and sustainable food choices in adults attending a diet clinic

**DOI:** 10.3389/fnut.2026.1983987

**Published:** 2026-10-28

**Authors:** Hilal Toklu Baloglu, Sevgi Karakose, Zeynep Yavuz, Hediye Unlu

**Affiliations:** Department of Nutrition and Dietetics, Faculty of Health Sciences, Bitlis Eren University, Bitlis, Türkiye

**Keywords:** carbon footprint, diet quality, Planetary Health Diet, sustainability, water footprint

## Abstract

**Background:**

Sustainable dietary patterns are increasingly recognized as essential for promoting both human and planetary health. Although the Planetary Health Diet was developed to integrate human health and environmental sustainability; empirical studies jointly evaluating PHDI adherence, estimated environmental footprints, and sustainable food-choice behaviors within the same population remain limited.

**Methods:**

This cross-sectional study evaluated adherence to the Planetary Health Diet and its relationships with diet quality, environmental footprints, and sustainable food choices among 300 adults aged 18–64 years attending a diet clinic. Dietary intake was assessed using a 24-h dietary recall. The Planetary Health Diet Index (PHDI), Healthy Eating Index-2020 (HEI-2020), carbon and water footprints, and Sustainable Food Choice Questionnaire (SUS-FCQ) scores were evaluated.

**Results:**

A moderate positive correlation was found between PHDI and HEI-2020 scores (*r* = 0.48, *p* < 0.001). Carbon (kg CO2 eq/d) and water footprints (L/d) were strongly correlated (*r* = 0.96, *p* < 0.001). Weak inverse correlations were observed between PHDI and both environmental footprint indicators (*r* = −0.13, *p* = 0.030). These associations remained weak but significant after multivariable adjustment for carbon (*β* = −0.105, *p* = 0.020) and water footprints (*β* = −0.102, *p* = 0.017), with similar findings in energy-standardized sensitivity analyses. Although environmental footprint values tended to decrease across PHDI quartiles, these differences were not statistically significant (*p* > 0.05). Higher SUS-FCQ scores were observed among women, individuals with higher educational attainment, and students and civil servants (*p* < 0.05).

**Conclusion:**

Higher PHDI scores were associated with improved diet quality and showed modest inverse associations with estimated environmental footprint indicators. Given that both indices were derived from the same dietary recall and include overlapping dietary components, this association should be interpreted cautiously. Sustainable food choice behaviors varied according to sociodemographic and lifestyle characteristics. These findings support integrated public health nutrition strategies that address both nutritional quality and environmental sustainability.

## 1 Introduction

Unhealthy dietary patterns are major contributors to the global burden of non-communicable diseases and are also increasingly recognized as important drivers of environmental degradation. According to the World Health Organization, obesity and overweight affect a substantial proportion of the global population and are associated with increased risk of chronic diseases such as type 2 diabetes, cardiovascular diseases, and certain cancers (1).

Beyond its health consequences, dietary patterns linked to obesity are also closely associated with environmental pressures. Food production systems contribute substantially to greenhouse gas emissions, land degradation, and water pollution (2). The Intergovernmental Panel on Climate Change has highlighted that climate change and its impacts on human health are accelerating, emphasizing the urgent need for sector-wide interventions, particularly within food systems (3). It is estimated that agriculture and food production are responsible for nearly 30% of global greenhouse gas emissions, 70% of freshwater use, and over 40% of land use (4).

Environmental impacts vary significantly across food groups. Clune et al. (5) demonstrated that plant-based foods such as fruits, vegetables, and grains have considerably lower greenhouse gas emissions compared to animal-based products, particularly red meat. Similarly, Mekonnen and Hoekstra (6) provided comprehensive assessments of water footprints across countries and food categories, highlighting substantial variations in resource use. The concept of a “sustainable diet” was first introduced by Gussow and Clancy (7), referring to dietary patterns that support both human health and environmental sustainability. Later, the FAO further defined sustainable diets as those with low environmental impact that contribute to food security and nutrition for present and future generations (8). Such diets typically emphasize plant-based foods while limiting processed, energy-dense, and animal-derived products, thereby supporting both ecological and health outcomes (9).

In 2019, the EAT–Lancet Commission proposed the Planetary Health Diet as a global reference model aimed at improving both human and environmental health (10). This dietary framework prioritizes plant-based foods—including fruits, vegetables, whole grains, legumes, and nuts—while recommending moderate consumption of animal-based foods and minimal intake of red and processed meats, refined grains, and added sugars (11). The widespread adoption of this dietary pattern, alongside improvements in food systems and reductions in food waste, is projected to substantially reduce greenhouse gas emissions and support sustainable food systems by 2050 (4).

According to a large-scale cohort study, adhering more closely to the Planetary Health Diet has been shown to significantly reduce environmental impacts—including greenhouse gas emissions and land use—while also contributing to improved health outcomes. Adherence to such dietary patterns is estimated to prevent deaths and certain types of cancer to a certain extent (12). Furthermore, adherence to the Planetary Health Diet has been associated with a reduced risk of cardiovascular disease (13). Furthermore, while inverse associations have been demonstrated between adherence to this diet and anthropometric measures such as body mass index and waist circumference (14), a reduction in the risk of metabolic syndrome and abdominal obesity has been reported (15). Furthermore, it has been shown that sociodemographic and behavioral factors influence adherence to dietary patterns (16). Digital food literacy has also emerged as another factor with potential importance in sustainable food choices; however, its relationship with adherence to the Planetary Health Diet requires further investigation (17–19).

Overall, the Planetary Health Diet represents a promising approach for promoting both individual and environmental health. Sustainable dietary patterns not only contribute to reducing environmental impacts but also play a key role in preventing chronic diseases. This study is based on a conceptual framework that assumes sustainable diets have simultaneous impacts on both human health and environmental outcomes. Accordingly, the PHDI was evaluated in relation to dietary quality (HEI-2020), environmental footprints (carbon and water), anthropometric indicators, and sustainable food choice behaviors. Although previous studies have examined several of these relationships separately, evidence simultaneously evaluating PHDI, HEI-2020, carbon and water footprints, and sustainable food choice behaviors within the same study remains limited.

This study aimed to provide a multidimensional evaluation of PHDI scores by examining their associations with diet quality, environmental footprints, anthropometric indicators, and sustainable food choice behaviors.

Based on existing evidence, we hypothesized that:

(1) Higher PHDI scores would be positively associated with HEI-2020 scores.
(2) Higher PHDI scores would be inversely associated with carbon and water footprints.
(3) Higher PHDI scores would be positively associated with sustainable food choice behaviors.
(4) Higher PHDI scores would be associated with more favorable anthropometric indicators (e.g., BMI and waist circumference).

## 2 Materials and methods

### 2.1 Sample selection

The sample size was calculated using the Raosoft sample size calculator based on a 95% confidence level, 5% margin of error, and an assumed response distribution of 50%, which provides the maximum sample size. The minimum required sample size was estimated as 245 participants (20). Since data losses were possible, 300 individuals were included in the study. This cross-sectional study was conducted among individuals aged 18–64 years attending the diet clinic of a public hospital in Bitlis, Türkiye, between January and July 2025. The clinic serves adults seeking nutritional counseling and is not restricted to a specific disease group. During the study period, individuals attending the clinic who met the eligibility criteria were informed about the study and invited to participate. Participation was voluntary, and individuals who agreed to participate and provided written informed consent were included in the study. Individuals with a diagnosed psychiatric disorder or eating disorder, pregnant or breastfeeding women, and those with conditions requiring significant dietary changes or restrictions (such as oncology, peptic ulcers, inflammatory bowel diseases, chronic kidney failure, or cirrhosis) were excluded from the study. Prior to the data collection process, ethical approval was obtained from the Bitlis Eren University Non-Interventional Clinical Research Ethics Committee (Decision No: 2024/9-5 Date: 02.01.2025). Additionally, all participants were provided with an explanation and asked to sign an informed consent form. The reporting of this cross-sectional study was guided by the STROBE statement.

### 2.2 Data collection

A general questionnaire was used to determine participants’ demographic characteristics and record their anthropometric measurements; a 24-h retrospective food consumption record was used to calculate the PHDI and carbon and water footprint; and the Sustainable Food Choice Questionnaire (SUS-FCQ) was used to assess sustainable food choices (21). The questionnaire forms were completed using a face-to-face interview technique. Participants’ anthropometric measurements—including weight, muscle, fat, and water percentages—were taken using a non-stretchable measuring tape and a portable precision scale (a device capable of measuring body composition). The Food and Nutrition Photo Catalog was utilized to accurately determine food quantities (22).

#### 2.2.1 Environmental footprint calculations

Data obtained from participants’ 24-h retrospective food consumption records were used in the carbon and water footprint calculations. Food consumption data were analyzed using the Nutrition Information System (BeBiS) program (23). The amount of each food consumed (g/day) was converted to kilograms and multiplied by the corresponding literature-based footprint coefficient. The environmental footprint coefficients were based on the compilation reported by Ceylan (24). Carbon-footprint coefficients (kg CO₂-eq/kg food) were derived from published life-cycle assessment data reported by Heller and Keoleian (25), Clune et al. (5) as compiled by Ceylan (24). Because the underlying studies differed in their system boundaries and production contexts, the coefficients did not represent a single harmonized life-cycle boundary. Therefore, the resulting carbon-footprint estimates were interpreted as comparative indicators rather than as standardized estimates covering an identical set of production, processing, transportation, retail, and consumption stages. Water-footprint coefficients were based on the Water Footprint Network methodology and data reported by Mekonnen and Hoekstra (6), as reported in Ceylan (24). Total water footprint represented the sum of green, blue, and gray water-footprint components. Water-footprint coefficients expressed as m^3^/ton are numerically equivalent to L/kg food. These coefficients were multiplied by the amount of each food consumed (kg/day), yielding participant-level water-footprint estimates in L/day.

When an exact food-specific coefficient was unavailable, the food was matched to the closest comparable food item or food category according to its main ingredient and food type. Where no reasonable equivalent was available, the item was not included in the respective footprint calculation. Participant-level daily carbon and water footprints were obtained by summing the contributions of all matched food items. The complete food-to-coefficient mapping, including the coefficients, original data sources, and substitutions used, is provided in Supplementary Table 1. Because Türkiye-specific environmental-footprint coefficients were not available for all foods, internationally published coefficients were used. Accordingly, the resulting carbon and water footprints should be interpreted as estimated comparative indicators rather than precise measures of the environmental impact of food production in Türkiye.

#### 2.2.2 Planetary Health Diet Index (PHDI)

The Planetary Health Diet Index (PHDI), developed by Cacau et al. (26) based on the EAT-Lancet reference diet, consists of 16 components and ranges from 0 to 150 points, with higher scores indicating greater adherence to the Planetary Health Diet. The index includes five adequacy components (nuts and peanuts, legumes, fruits, total vegetables, and whole grains), five optimum components (eggs, fish and seafood, tubers and potatoes, dairy, and unsaturated oils), two ratio components (dark green vegetables to total vegetables and red and orange vegetables to total vegetables), and four moderation components (red meat, chicken and substitutes, animal fats, and added sugars). Component scoring is based on caloric density relative to total energy intake, according to the cutoff values proposed by Cacau et al. (26). Most components contribute a maximum of 10 points, whereas the two ratio components contribute a maximum of 5 points each.

#### 2.2.3 Healthy Eating Index-2020 (HEI-2020)

The Healthy Eating Index was first introduced to the literature by the U.S. Department of Agriculture (USDA) Center for Nutrition Policy and Promotion (27). The new version of the Healthy Eating Index (HEI) (2020) is designed to use a scoring system based on 1,000 calories per day. It is thus used to assess diet quality independently of diet quantity. Scores for all components except sodium are based on recommendations for 1,200–2,400 kcal (the sodium recommendation is capped at 2,300 mg). Minimum values are based on the amount per 1,000 kcal for total fruits, whole fruits, total vegetables, leafy green vegetables and legumes, whole grains, dairy products, total protein-rich foods, seafood, and plant-based proteins. If these foods are not consumed at all, the score is zero points. However, some components (fatty acids, refined grains, sodium, added sugars, and saturated fats) do not have a specified minimum value; consuming them in small amounts increases the score. For example, consuming saturated fats at ≤8% of total energy yields 10 points, while consumption of ≥16% results in zero points. In the assessment, diets with high quality (80 or higher) are grouped as good, those with moderate quality (needs improvement) are 51–80, and poor (inadequate) are 50 or below (28).

#### 2.2.4 Sustainable Food Choice Questionnaire (SUS-FCQ)

The Sustainable Food Choice Questionnaire (SUS-FCQ), developed by Verain et al., was used to assess sustainability-related food-choice considerations. The instrument was designed to capture sustainability aspects that are not sufficiently represented in conventional food-choice motivation measures such as the Food Choice Questionnaire (FCQ). It comprises 16 items grouped into two dimensions: General Sustainability, which includes 13 items addressing environmental, ethical, and animal-welfare considerations, and Local and Seasonal, which consists of three items. Each item is rated on a seven-point Likert scale ranging from 1 (strongly disagree) to 7 (strongly agree), and dimension scores are obtained by summing the corresponding item responses. In the original study, Cronbach’s alpha was 0.962 for General Sustainability and 0.853 for Local and Seasonal (29). The Turkish adaptation was validated by Özkaya in 2023, with Cronbach’s alpha coefficients of 0.971 for General Sustainability, 0.866 for Local and Seasonal, and 0.961 for the total SUS-FCQ score (21).

### 2.3 Statistical analysis of the data

All statistical analyses were performed using SPSS for Windows version 27.0, and *p* < 0.05 was considered statistically significant. Descriptive statistics were presented as number (*n*), percentage (%), mean ± standard deviation, or median (25th–75th percentile), as appropriate. The normality of the scale scores was checked using the Kolmogorov–Smirnov test. Carbon and water footprint values did not show a normal distribution; therefore, these variables were log-transformed before further analyses. BMI, waist circumference, and body fat percentage were considered as secondary outcomes to examine their relationships with dietary sustainability. MANCOVA was used to evaluate whether PHDI, HEI-2020, and environmental footprint measures differed according to sociodemographic and lifestyle characteristics. Since the amount of food consumed may affect both dietary sustainability indicators and environmental footprints, total energy intake was included in the model as a covariate.

Linear regression models were used to examine the relationship between PHDI and environmental footprints. In the baseline models, after applying a logarithmic transformation, carbon and water footprints were analyzed as dependent variables. Age, gender, education level, BMI, physical activity, smoking status, and total energy intake were included in the model as covariates. Additional analyses were also conducted using energy-standardized (per 1,000 kcal) carbon and water footprints. The same variables were used in these models, with the exception that total energy intake was not included. PHDI was added to the second block, and changes in explained variance (Δ*R*^2^) were examined.

The Mann–Whitney *U* test was used to compare SUS-FCQ scores between two groups, while the Kruskal–Wallis test was used for comparisons involving more than two groups. When a significant difference was found, Bonferroni *post hoc* comparisons were performed to identify the specific groups between which the difference existed.

## 3 Results

Table 1 presents the participants’ demographic characteristics, as well as their lifestyle and anthropometric characteristics. The study included 300 participants, 51% of whom were women and 49% men. The mean age was 38.3 ± 13.6 years; approximately half of the participants (51%) were of normal weight, 17% were overweight, and 31% were obese. Thirty-nine percent had completed elementary or middle school, while 31% had a bachelor’s or associate’s degree. The vast majority of participants (69%) were married, and housewives (32 percent) and public sector employees (24%) constituted the most common occupational groups.

**Table 1:** Demographic, lifestyle, and anthropometric characteristics of the participants.

Variable	*n* (%)
Gender	
Male	148 (49)
Female	152 (51)
Educational status	
Illiterate	36 (12)
Elementary–Middle school graduate	117 (39)
High school graduate	55 (18)
Bachelor’s/Associate’s degree graduate	92 (31)
Marital status	
Single	93 (31)
Married	207 (69)
Occupation	
Student	39 (13)
Self-employed	70 (23)
Civil servant	72 (24)
Laborer	14 (5)
Retiree	8 (3)
Housewife	97 (32)
BMI category	
Normal weight	154 (51)
Overweight	52 (17)
Obesity	94 (31)
Physical activity/exercise status	
Yes	74 (25)
Sometimes	57 (19)
No	169 (56)
Tobacco use	
No	202 (67)
Yes	98 (33)
Alcohol use	
Never	285 (95)
Rarely	15 (5)
Anthropometric measurements	Mean ± SD
Weight (kg)	76.9 ± 14.0
BMI (kg/m^2^)	27.8 ± 5.5
Waist circumference (cm)	92.5 ± 14.0
Hip circumference (cm)	106.5 ± 11.0
Upper arm circumference (cm)	30.8 ± 3.9
Body fat (%)	26.3 ± 10.2
Muscle mass (%)	53.7 ± 9.8
Body water (%)	52.9 ± 9.4
Age (years)	38.3 ± 13.6

Regarding lifestyle characteristics, 56% of the participants did not engage in regular physical activity, 33% were smokers, and 95% did not consume alcohol. The mean BMI was 27.8 ± 5.5 kg/m^2^, mean waist circumference was 92.5 ± 14.0 cm, and mean body fat percentage was 26.3 ± 10.2%.

Table 2 presents the correlations between environmental footprints, PHDI, HEI-2020, sustainable food-choice scores, and anthropometric indicators. Carbon and water footprints were strongly positively correlated (*r* = 0.96, *p* < 0.001). PHDI showed weak inverse correlations with both carbon and water footprints (*r* = −0.13, *p* < 0.05) and a moderate positive correlation with HEI-2020 (*r* = 0.48, *p* < 0.001). No significant correlations were observed between PHDI and SUS-FCQ total score, BMI, waist circumference, or body fat percentage (*p* > 0.05). SUS-FCQ was weakly inversely correlated with waist circumference (*r* = −0.13, *p* < 0.05).

**Table 2:** Correlations between carbon and water footprint, PHDI, HEI-2020, and SUS-FCQ total scores.

Variable	Carbon footprint	Water footprint	PHDI	HEI-2020	SUS-FCQ	BMI	Waist circumference	Body fat %
Carbon footprint	1	0.96^**^	−0.13^*^	−0.09	0.01	0.08	0.10	0.09
Water footprint		1	−0.13^*^	−0.08	−0.01	0.06	0.10	0.08
PHDI			1	0.48^**^	−0.01	0.01	−0.02	0.09
HEI-2020				1	−0.02	0.08	0.05	0.13^*^
SUS-FCQ					1	−0.07	−0.13^*^	0.01

Values are Pearson correlation coefficients (*r*). ^*^p < 0.05; ^**^p < 0.001. PHDI, Planetary Health Diet Index; HEI-2020, Healthy Eating Index-2020; SUS-FCQ, Sustainable Food Choice Questionnaire; BMI, body mass index. Log transformation was applied to the carbon and water footprint variables before analysis.

Table 3 presents the results of a multivariate covariance analysis examining the effects of sociodemographic and lifestyle variables on the PHDI, HEI-2020, carbon footprint, and water footprint. According to the results, daily computer/television usage time, educational status, gender, and occupation had no statistically significant effect on the dependent variables (*p* > 0.05). Although a marginal trend of association was observed in the multivariate analysis for physical activity level, this relationship was not found to be statistically significant (*p* = 0.058).

**Table 3:** Multivariate covariance analysis for PHDI, HEI-2020, carbon, and water footprint according to sociodemographic and lifestyle variables.

Variable	Wilks’ *λ*	*F*	*p*	Partial *η*^2^
Daily computer/TV usage time	0.963	0.935	0.511	0.013
Physical activity status	0.950	1.895	0.058	0.025
Educational status	0.969	0.768	0.684	0.010
Gender	0.989	0.853	0.493	0.011
Occupation	0.924	1.164	0.278	0.020

The energy variable was included in the model as a covariate for multivariate covariance analysis (MANCOVA). Log transformation was applied to the carbon and water footprint variables before analysis; PHDI: Planetary Health Diet Index, HEI-2020: Healthy Eating Index-2020.

Table 4 presents a comparison of total scores on the SUS-FCQ and its subscales by sociodemographic variables. The total score of the scale showed significant differences based on gender, educational status, occupation, BMI category, and physical activity level (*p* < 0.05). It was found that women had higher sustainable nutrition SUS-FCQ total scores than men. It was observed that sustainable food choice scores increased with higher education levels, particularly among college and associate degree graduates. Among occupational groups, civil servants and students were found to have higher scores compared to some other occupational groups. When BMI categories were examined, it was found that the scores of individuals classified as overweight were higher than those of individuals with obesity. Additionally, it was observed that individuals who engaged in physical activity had higher sustainable food choice scores. In the local-seasonal subdimension, a significant difference was identified only based on physical activity.

**Table 4:** Comparison of Sustainable Food Choice Questionnaire (SUS-FCQ) and subscale scores according to sociodemographic variables.

Variable	SUS-FCQ total (25–75th percentile)	*p*	Overall sustainability (25–75th percentile)	*p*	Local-seasonal (25th–75th percentile)	*p*
Gender		0.015		0.009		0.825^*^
Male	95 (87–102)^a^		76 (67.5–82.5)^a^		21 (19–21)	
Women	98 (90–106)ᵇ		78.5 (72–87)ᵇ		21 (19–21)	
Educational status		<0.001		<0.001		0.876^**^
Illiterate	96 (90–103.5)^a^		77 (71.5–82.5)ᵃ		21 (18–21)	
Elementary–middle school	93 (85–98)ᵃ		73 (66–79)^a^		21 (19–21)	
High school	98 (90.5–103.5)ᵃᵇ		79 (71.5–84)ᵃᵇ		21 (19–21)	
Undergraduate and associate’s degree	102 (96.5–107.5)ᵇ		82 (76.5–88)^b^		21 (19–21)	
Vocational		<0.001		<0.001		0.212^**^
Student	100 (97–105.5)ᵇ		81 (76–86)ᵇ		21 (20–21)	
Self-employed	93.5 (84–101)ᵃ		73 (65–80)^a^		21 (19–21)	
Civil servant	101 (94.5–106)ᵇ		81 (75.5–86)ᵇ		21 (19–21)	
Worker	90 (85–95)ᵃ		70.5 (64–74)ᵃ		19.5 (19–21)	
Retiree	84.5 (76–98)^a^		71.5 (56.5–78.5)^a^		20 (16.5–21)	
Housewife	96 (89–103)ᵃᵇ		77 (69–84)ᵃᵇ		21 (18–21)	
BMI category		0.021		0.033		0.056^**^
Normal	97.5 (89–105)^ab^		78 (70–85)^ab^		21 (19–21)	
Overweight	99 (92.5–105.5)^b^		80.5 (74.5–85.5)^b^		21 (19–21)	
Obesity	95 (86–101)^a^		76 (69–82)^a^		21 (18–21)	
Physical activity		0.031		0.030		0.026^**^
Yes	97 (91–102)^ab^		78 (72–83)^ab^		20 (18–21)ᵃ	
Sometimes	99 (95–109)ᵇ		80 (74–89)ᵇ		21 (18–21)ᵇ	
No	96 (87–103)ᵃ		77 (68–83)ᵃ		21 (19–21)ᵇ	

^*^Mann–Whitney *U* test, ^**^Kruskal-Wallis test, SUS-FCQ, Sustainable Food Choice Questionnaire. Different superscript letters (a, b) indicate statistically significant differences between groups based on Bonferroni-adjusted post hoc comparisons; groups sharing the same letter are not significantly different.

Table 5 presents environmental footprint and Healthy Eating Index values according to PHDI quartiles. No significant difference was found between groups in terms of carbon footprint (*p* = 0.216) and water footprint (*p* = 0.227). However, it was determined that individuals in the fourth quartile, where the PHDI score was highest, had higher Healthy Eating Index scores compared to the other quartiles (*p* < 0.001).

**Table 5:** Environmental footprint and Healthy Eating Index values by PHDI quartiles.

Variable	PHDI				*F*	*p*
	Q1 (*n* = 75)	Q2 (*n* = 75)	Q3 (*n* = 75)	Q4 (*n* = 75)		
Carbon footprint	0.338 ± 0.170	0.292 ± 0.153	0.287 ± 0.162	0.287 ± 0.205	1.495	0.216
Water footprint	3.181 ± 0.163	3.152 ± 0.137	3.132 ± 0.143	3.138 ± 0.179	1.454	0.227
HEI-2020	32.79 ± 8.24	37.44 ± 9.44	38.77 ± 9.39	44.59 ± 10.52	19.923	<0.001

Log transformation was performed on the carbon and water footprint variables prior to analysis. PHDI, Planetary Health Diet Index, HEI-2020, Healthy Eating Index-2020.

In multivariable analyses (Table 6), higher PHDI scores were weakly but significantly associated with lower absolute carbon and water footprints after adjustment for age, sex, education, BMI, physical activity, smoking status, and total energy intake. PHDI was inversely associated with both carbon footprint (*β* = −0.105, *p* = 0.020) and water footprint (*β* = −0.102, *p* = 0.017). The addition of PHDI accounted for a small additional proportion of the variance in carbon (Δ*R*^2^ = 0.011, *p* = 0.020) and water footprints (ΔR^2^ = 0.010, *p* = 0.017). These associations remained significant in sensitivity analyses using energy-standardized footprints per 1,000 kcal, with PHDI showing inverse associations with both carbon (*β* = −0.125, *p* = 0.031) and water footprints (*β* = −0.127, *p* = 0.028). However, the additional variance explained by PHDI remained small (ΔR^2^ = 0.015 and 0.016, respectively).

**Table 6:** Multivariable associations of PHDI with absolute and energy-standardized environmental footprints.

Outcome	PHDI *β*	*p*	Model *R*^2^	Δ*R*^2^	*p* (Δ*R*^2^)
Absolute carbon footprint (log-transformed)	−0.105	0.020	0.420	0.011	0.020
Absolute water footprint (log-transformed)	−0.102	0.017	0.478	0.010	0.017
Carbon footprint/1000 kcal (log-transformed)	−0.125	0.031	0.046	0.015	0.031
Water footprint/1000 kcal (log-transformed)	−0.127	0.028	0.043	0.016	0.028

*β*, standardized regression coefficient; Δ*R*^2^, change in explained variance after the addition of PHDI to the covariate-adjusted model. Absolute footprint models were adjusted for age, sex, education, BMI, physical activity, smoking status, and total energy intake. Energy-standardized sensitivity models (per 1,000 kcal) were adjusted for age, sex, education, BMI, physical activity, and smoking status. Environmental footprint outcomes were log-transformed before regression analyses.

## 4 Discussion

This study aimed to evaluate the relationships between the Planetary Health Diet Index, the Healthy Eating Index, sustainable food choices, and carbon and water footprint indicators in adults. Previous studies have examined PHDI in relation to diet quality, environmental outcomes, or anthropometric indicators; however, fewer studies have evaluated these dimensions together with self-reported sustainable food-choice behaviors within the same population. Therefore, the contribution of the present study lies primarily in the joint evaluation of nutritional quality, estimated environmental footprints, sustainable food-choice behaviors, and anthropometric indicators rather than in demonstrating the expected alignment between PHDI and established diet-quality indices. According to our results, the Planetary Health Diet score is positively associated with healthy eating. When evaluated using the carbon and water footprints, a weak but negative correlation was also observed, with an inverse trend. No significant association was observed between PHDI and sustainable food-choice scores or anthropometric indicators, although sustainable food-choice scores differed according to certain sociodemographic characteristics and were associated with waist circumference. These results indicate that sustainable dietary behaviors should be evaluated not only from an environmental perspective but also in terms of dietary quality and individual health status.

The Planetary Health Diet Index (PHDI) integrates health and sustainability goals (30). While low compliance with this index is associated with poor dietary habits, (31) higher scores have been linked to an improvement in overall diet quality (31). Kendel et al. demonstrated that planetary diet alignment is associated with diet quality (32). Additionally, the Planetary Health Diet is associated with a lower risk of micronutrient deficiency (with a strong inverse relationship) (33). While the Healthy Eating Index measures dietary quality, the Planetary Health Diet Index (PHDI) is proposed as a more comprehensive index that evaluates both nutritional quality and environmental sustainability (30). On the other hand, sustainability-focused dietary recommendations such as the PHDI share similar cross-sectional associations with cardiometabolic risk as the HEI-2015 (34). Consistent with previous studies, higher PHDI scores were associated with higher diet quality in the present study. This finding was expected given that both the PHDI and HEI-2020 emphasize several health-promoting dietary components and should therefore be regarded as confirmatory rather than as evidence of a novel relationship. However, this association should be interpreted cautiously because both indices were calculated from the same 24-h dietary recall and share several conceptually overlapping components, including fruits, vegetables, whole grains, plant-based protein sources, added sugars, and aspects of fat quality. Therefore, the observed correlation may partly reflect the conceptual and mathematical overlap between the two scoring systems rather than an independent relationship between planetary diet adherence and diet quality.

Current food systems are pushing many environmental impact thresholds to the limit, and a dietary transition toward sustainable nutrition models is needed. Identifying policies to promote this dietary transition requires accurate information on the environmental impact of dietary choices (35). It is becoming increasingly important to prioritize sustainable dietary habits that promote health and have minimal environmental impacts; understanding the impact of food production activities and population dietary habits on water use is recommended for sustainable water management (36). A better level of adaptation to the planetary diet is associated with effective land use in greenhouse gas emissions, as well as preventing 10–39% of deaths and cancers (37). In a cohort study, the PHDI was similarly associated with lower all-cause mortality rates and demonstrated a comparably low environmental impact (38). It has been shown that higher diet quality is associated with lower diet-related greenhouse gas emissions (30). However, regarding estimated environmental impacts, it is also noted that higher adherence to the planetary diet reduces greenhouse gas emissions but increases the total water footprint and land use (39). Additionally, the indices and guidelines used in studies also reveal the relationship between water and carbon footprints and the Planetary Health Diet in various ways (35, 40). Therefore, changes in dietary habits may offer potential benefits for both the environment and health. However, there is uncertainty regarding the magnitude of these effects and the dietary changes required to achieve them (41). Dietary composition, different production methods of foods, processing, and portion sizes may influence environmental impacts (42). In our study, PHDI showed weak inverse associations with carbon and water footprints, which remained significant after multivariable adjustment and in energy-standardized sensitivity analyses. However, PHDI explained only a small additional proportion of variance, and differences across PHDI quartiles were not significant, indicating that these associations were modest.

The Planetary Health Diet and other sustainable diets are beneficial for both people and the environment and are associated with a reduced risk of overweight and obesity. A study by Cacau et al. (26) found that individuals demonstrating higher adherence to the Planetary Health Diet Index had not only lower BMI but also smaller waist circumference and lower rates of abdominal obesity. It is emphasized that as adherence to the Planetary Health Diet increases, both BMI, waist circumference, and body fat measurements decrease (43, 44). However, while an inverse association was found in the elderly, the significant effect disappeared after adjusting for confounding factors (45). On the other hand, other studies (31, 46) have shown higher adherence to sustainable dietary patterns among individuals with overweight or obesity. In this study, no significant association was found between the Planetary Health Diet score and BMI, body fat percentage, or waist circumference. Anthropometric indicators are influenced by multiple behavioral, metabolic, and lifestyle-related factors beyond dietary quality alone, which may partly explain the absence of significant associations in this cross-sectional analysis. However, a significant association was found between the total score for sustainable food choices and waist circumference. This finding suggests that behaviors reflecting specific food choices may exhibit more specific relationships with abdominal adiposity compared to general dietary indices. Thus, our findings are directionally consistent with studies reporting lower environmental impacts with greater PHDI adherence, but the small effect sizes and non-significant quartile differences indicate a substantially more modest association in this clinic-based sample. Differences in food-production systems, footprint databases, and dietary assessment methods may partly account for variation across studies.

Guiding the population toward a healthy plant-based diet could be a potential goal for preventing and managing chronic diet-related diseases and ensuring future food security (47). Studies conducted in this regard indicate that individuals’ dietary preferences are influenced not only by individual factors but also by sociodemographic characteristics. It is necessary to consider the characteristics of those who adopt healthier and more environmentally friendly dietary patterns (48). Women place greater importance on a healthier, plant-based diet; they consume less red and processed meat, while their fruit and vegetable intake is higher (29). In a cohort study, it was reported that adherence to a sustainable healthy diet increased over a 20-year period without a corresponding decrease in greenhouse gas emissions; however, BMI also increased during this period (49). Negative associations between sustainable diets and BMI have been demonstrated (50, 51), and a systematic review found that studies yielded inconsistent results (52). Additionally, low levels of physical activity are associated with poor dietary habits (53). The literature indicates that educational level is an important determinant of sustainable dietary behaviors. Furthermore, it has been shown that higher-educated groups exhibit healthier, yet equally environmentally sustainable dietary behaviors compared to lower-educated groups, and that women demonstrate healthier dietary habits than men (54). Scores for sustainable food choices showed significant differences according to sociodemographic variables. The complex relationships observed between higher scores in women and individuals with higher education levels and BMI, and physical activity indicate that sustainable dietary behaviors are shaped by multidimensional lifestyle factors. In contrast to the expected PHDI–HEI-2020 association, the absence of an association between PHDI and SUS-FCQ provides a less intuitive finding. It suggests that adherence to a dietary pattern designed around health and environmental targets does not necessarily correspond to individuals’ self-reported motivations and behaviors related to sustainable food choice. While the PHDI is based primarily on dietary intake, the SUS-FCQ reflects broader considerations related to food-choice behaviors. Thus, these measures may capture distinct dimensions of sustainable eating. Digital food literacy was not directly assessed in the present study. Future studies using validated measures should examine whether digital food literacy is associated with sustainable food choices and Planetary Health Diet adherence.

### 4.1 Strengths and limitations

One of the strengths of this study is its contribution to the limited evidence jointly evaluating the Planetary Health Diet Index, environmental footprints (carbon and water), and sustainable food choice behaviors in adults. Additionally, the joint analysis of anthropometric measurements with nutrition and sustainability indicators contributes to a multidimensional assessment of the subject. Our study also has limitations. A 24-h food consumption record was used in the PHDI, HEI-2020, and carbon and water footprint calculations. Some of the correlations among these measures may stem from shared baseline dietary data. The 24-h dietary recall, however, may also be an assessment that reflects only intake on the day of the assessment and does not fully reflect overall dietary habits. Furthermore, the fact that participants were selected from a nutrition clinic at a local hospital limits the generalizability of the findings. Although relevant covariates were used in the multivariate model analyses, our results may have been influenced by some residual confounding effects. Finally, the water and carbon footprint coefficients were compiled from various sources in the literature; therefore, the obtained values should be interpreted as comparative estimates rather than standardized absolute environmental impacts. Furthermore, digital food literacy was not directly assessed in this study; therefore, its potential role in shaping sustainable food choices and adherence to the Planetary Health Diet could not be evaluated. Future studies incorporating validated measures of digital food literacy may provide a more comprehensive understanding of these relationships. In addition, the environmental impact factors used in the carbon and water footprint calculations were obtained from international data sources. Due to Turkey-specific production systems, agricultural practices, and supply chain differences, these coefficients may not fully reflect the actual environmental impacts in Turkey. Therefore, the carbon and water footprint values obtained should be interpreted as estimated indicators for comparing individuals’ environmental impacts rather than absolute values.

## 5 Conclusion

This study identified a positive association between PHDI and HEI-2020 scores, although their shared dietary data source and overlapping components should be considered. Higher PHDI scores were weakly associated with lower estimated carbon and water footprints after multivariable adjustment and in energy-standardized sensitivity analyses. Overall, these findings suggest modest associations between PHDI, diet quality, and environmental footprint indicators. Therefore, it is important to address dietary quality and environmental sustainability together in public health policies. Public health interventions targeting sustainable dietary behaviors may contribute to both nutritional improvement and environmental sustainability.

## Data Availability

The raw data supporting the conclusions of this article will be made available by the authors, without undue reservation.
